# Adult Chiari Type I Malformation Presenting as Emergent Acute Respiratory Insufficiency: A Case Report and Literature Review

**DOI:** 10.7759/cureus.81898

**Published:** 2025-04-08

**Authors:** Pedro Ribeiro, João Nogueira, Maura Cambango, Leandro Oliveira, Frederica Coimbra

**Affiliations:** 1 Neurosurgery, Hospital de Braga, Braga, PRT

**Keywords:** abrupt onset, acute respiratory insufficiency, chiari i malformation, coma, emergent surgery, syringomyelia

## Abstract

Chiari type I malformation (CM-1) is a condition that is often asymptomatic, though when symptoms occur, they commonly involve occipital headaches. Acute respiratory insufficiency as the presenting symptom requiring emergent surgical intervention is extremely rare. We present the case of a 51-year-old female who developed sudden-onset respiratory insufficiency due to CM-1 and syringomyelia affecting the brainstem, necessitating urgent surgical intervention. The patient, with a history of mild tetraparesis, arrived at the emergency department with an acute loss of consciousness, severe respiratory acidosis, and acidemia. Imaging revealed tonsillar herniation at the foramen magnum, significant compression of the bulbomedullary junction, and an extensive syringomyelic cavity extending from the cervical to the dorsal spine. The patient underwent a comprehensive evaluation, with no alternative causes found for the clinical presentation. An emergent posterior fossa decompressive craniectomy and C1 laminectomy were performed, leading to a satisfactory clinical recovery. This case highlights the importance of prompt surgical intervention in rare presentations of CM-1, which can lead to significant improvement in symptoms and favorable outcomes.

## Introduction

Chiari type I malformation (CM) is a relatively common condition identified in approximately 1% of patients undergoing magnetic resonance imaging (MRI) [1,2]. Despite its prevalence, the underlying pathophysiological mechanisms remain poorly understood [3]. While CM is often asymptomatic, symptomatic cases typically present with gradually progressive symptoms [1,4]. The most frequent clinical manifestation is effort-induced headache [1]. Acute respiratory insufficiency as an initial presentation of CM is exceedingly rare, with only a few cases documented in the literature [5-12]. This case report aims to present a rare clinical case of an adult patient with abrupt-onset respiratory insufficiency due to CM, necessitating emergent surgical intervention, and to provide a literature review of similar cases involving acute respiratory insufficiency as the presenting symptom requiring urgent surgical treatment.

## Case presentation

A 51-year-old female presented to the emergency department following an acute loss of consciousness. Prior to this event, the patient reported a progressive global decrease in limb strength, without any specific neurological deficits, and exhibited hyperreflexia in her osteotendinous reflexes. Her overall functional status was assessed using the Modified Rankin Scale (mRS), where she scored 2. Due to these findings, she was referred for further neurological evaluation and was awaiting cervical magnetic resonance imaging (MRI).

**Figure 1:**
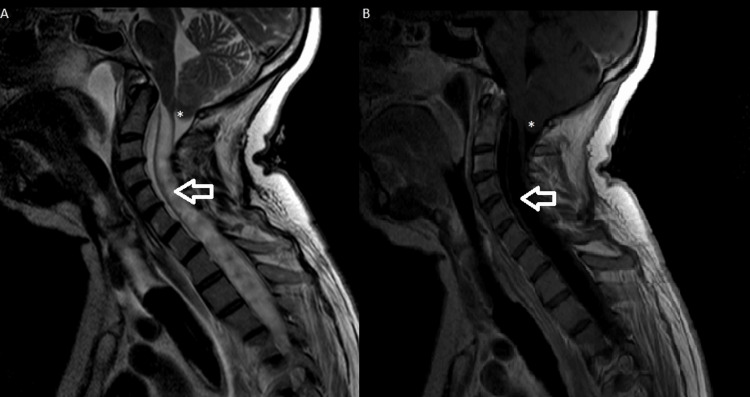
Magnetic resonance imaging, T2-weighted (A) and T1-weighted (B), performed at diagnosis before surgery. Cerebellar tonsillar herniation at the foramen magnum, significantly compressing the bulbo-medullary junction (white asterisk), with an extensive syringomyelic cavity extending through the cervical and dorsal spine (white arrow).

The patient was found comatose by her family and was immediately transported to the emergency department of her reference hospital. Upon arrival, she scored 6 on the Glasgow Coma Scale and demonstrated severe respiratory acidosis with acidemia, confirmed by arterial blood gas analysis (fraction of inspired oxygen (FiO2) 80%: pH 6.9; partial pressure of carbon dioxide (pCO2) 127 mmHg; partial pressure of oxygen (pO2) 220 mmHg). A computed tomography (CT) scan of the brain revealed a cerebellar tonsillar herniation with syringomyelia. Subsequently, a cervical and dorsal MRI (Figure 1) showed a tonsillar herniation at the foramen magnum, significantly compressing the bulbo-medullary junction, with an extensive syringomyelic cavity extending through the cervical and dorsal spine.

The most common causes, such as stroke, metabolic disturbances, and seizures, were excluded. Given the severity of her condition, the patient was transferred to a hospital with a neurosurgery unit. An emergency decompressive suboccipital median craniectomy and C1 laminectomy with dural opening and duroplasty were performed.

Post-surgery, the patient was admitted to an intensive care unit (ICU), where she required invasive mechanical ventilation and orotracheal intubation for two weeks. Following tracheostomy and ventilatory weaning, she recovered to a state of normal consciousness and was cooperative and oriented, and she remained in the ICU due to persistent hypercapnia, particularly at night, necessitating continuous nocturnal bilevel positive airway pressure (BIPAP). She remained in this unit for approximately two additional months before being transferred to a rehabilitation unit.

**Figure 2:**
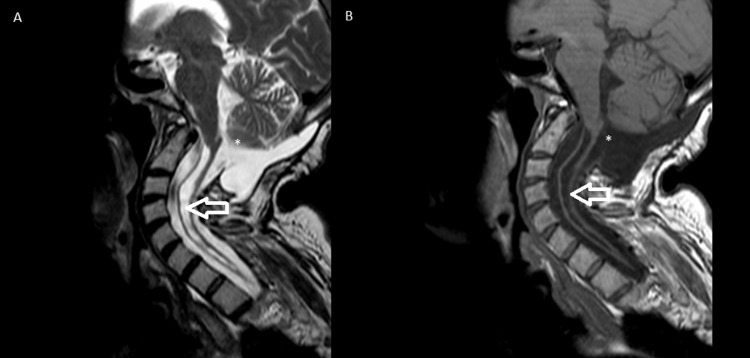
Magnetic resonance imaging, T2-weighted (A) and T1-weighted (B), performed 1 year after surgery Postoperative changes following foramen magnum decompression (white asterisk) and reduction of the syringomyelic cavity previously observed in the preoperative scan (white arrow)

The patient underwent an intensive rehabilitation program for three months, gradually regaining functional capacity. At her one-year follow-up, she continued to experience mild tetraparesis and required nocturnal mechanical ventilation with BIPAP. Her overall functional status was assessed using the mRS, where she scored 3, indicating she was unable to perform all previous activities but was able to carry out daily activities with some limitations. The MRI scans of the brain and cervical spine, taken approximately one year after the event, are presented in Figure 2 and show that the volume of the extensive syringohydromyelic cavity is reduced in the current examination, with a consequent reduction in the volume occupied by the observable portions of the spinal cord.

## Discussion

Eight articles from the literature search were included, covering the period from 1988 to 2017. In total, 10 adult patients with CM-1 presented with an abrupt onset of acute respiratory insufficiency, requiring emergent surgical treatment. The main characteristics of the 11 reported patients (including our clinical case) are summarized in Table 1.

**Table 1:** Summary of characteristics of 11 reported patients with Chiari type I malformation and acute respiratory insufficiency requiring emergent surgery

Author	Year	Age (years)	Gender	Previous Symptoms	Chiari Type	Syringomyelia/Syringobulbia	Surgery	Outcome
Bullock et al. [5]	1988	26	Female	Dyspnea for two weeks	I	Yes	Decompressive suboccipital craniectomy and duroplasty	Nighttime mechanical ventilation
Fish et al. [6]	1988	63	Female	Ataxia, occipital pain and dyspnea 10 days before, asthenia, dysphagia	I	-	Decompressive suboccipital craniectomy and C1 laminectomy	Asymptomatic
Fish et al. [6]	1988	54	Female	Ataxia, respiratory pauses, dysphagia, tetraparesis	I	-	Decompressive suboccipital craniectomy and C1 laminectomy	Ataxia and tetraparesis
Alvarez et al. [7]	1995	38	Male	Nocturnal dyspnea	I	Yes	Decompressive suboccipital craniectomy and C1 and C2 laminectomy	Resolution of severe symptoms
Omer et al. [8]	1996	23	Male	Paraparesis	I	Yes	Decompression of the foramen magnum	Asymptomatic
Omer et al. [8]	1996	26	Female	Dyspnea, dysphagia, asthenia, paraparesis	I	No	Decompression of the foramen magnum	Sudden death 2 months after surgery
Gentry et al. [9]	2001	38	Male	Dyspnea, dysphagia, ataxia, upper limb weakness, daytime hypersomnolence, snoring	I	Yes	Decompressive suboccipital craniectomy and C1, C2 and C3 laminectomy	Resolution of severe symptoms
Tsara et al. [10]	2005	32	Male	Morning headache, fatigue	I	Yes	Decompression of the foramen magnum and duroplasty	Persistence of sleep-related respiratory changes, daytime hypercapnia
Gladding et al. [11]	2005	22	Male	Difficulty breathing at night	I	Yes	Decompression of the foramen magnum and C1 and C2 laminectomy	Asymptomatic
Vasani et al. [12]	2017	35	Male	Dyspnea	1.5	Yes	Decompressive suboccipital craniectomy and C1 and C2 laminectomy	Asymptomatic
Ribeiro et al.	2024	51	Female	Mild tetraparesis	1.5	Yes	Decompressive suboccipital craniectomy and C1 laminectomy	Mild tetraparesis; nighttime mechanical ventilation

The average age was 37.1 years (range 22-63 years), with a slight male predominance (n=6, 54.5%). All patients presented with acute respiratory insufficiency. Among the 11 included patients, 8 had prior complaints of dyspnea. Regarding the presence of syringomyelia/syringobulbia, this was documented in 8 out of the 11 patients. Decompressive surgery was performed on all patients. One case of mortality was reported among the 11 included patients, which occurred 2 months after surgery. The outcomes of the included patients are provided in Table 1.

CM-1 is often a chronic and asymptomatic condition. Many cases of CM-1 are found incidentally on MRI or CT scans [13]. Presentation as a life-threatening acute condition requiring emergent treatment is very rare. Only 4.6% of patients with CM-1 require urgent decompression surgery (within 24 hours of symptom deterioration) [4]. In the limited literature available on acute presentation in patients with CM-1, there is a significant association with preceding traumatic events, approximately 41.5% [14]. In patients with acute presentation, sudden death is estimated to occur in 12% of cases [14]. In this study, we focused on patients with CM-1 who presented acutely, without associated trauma, and with spontaneous symptoms in the absence of an apparent causal factor.

From an anatomical and imaging standpoint upon admission, we observed that in the vast majority of published clinical cases, as in our own, patients who presented more acutely had associated syringomyelic cavities. This finding aligns with the literature, which shows that patients with acute presentations tend to have larger and more extensive syrinx cavities compared to those with non-acute cases [4]. Massimi et al., in their review of patients with Chiari type I malformation and acute presentation, found syringomyelia in approximately 51% of cases [14]. Despite these findings, it is important to note that the prevalence of syringomyelia in the general population is relatively high, and in the vast majority of cases, surgery is not required [15]. Despite the association of CM-1 with syringomyelia, the natural history of this association appears to be benign, and most conservatively treated patients will not require surgery [16].

Clinically, previously published cases of acute CM-1 presentations most often involved spinal cord signs and symptoms, vocal cord paralysis, acute respiratory distress, cranial nerve dysfunction, or syncope [17]. Our clinical case aligns with this evidence. Follow-up of the patients selected for this study indicates that, in general, they had an excellent outcome, with symptom resolution before the event in almost all cases.

The type of surgery used in the collected cases was primarily decompressive surgery with or without cervical laminectomy, following standard surgical procedures. Despite the various surgical techniques available for the treatment of this condition [18,19], there is insufficient evidence to suggest that one specific type of surgical intervention is superior to another in the case of an acute symptomatic presentation.

Patients with CM-1 who have mild symptoms and a long follow-up period and are undergoing conservative treatment generally improve or do not worsen, with acute adverse events being infrequent [20]. Identifying risk factors for the acute deterioration of patients with Chiari type I malformation would facilitate the decision to proceed with early surgery. However, based on the available evidence, it remains very challenging to determine risk factors for the sudden deterioration of these patients [20].

## Conclusions

In conclusion, our case contributes to the growing, although still limited, body of literature on acute presentations of CM-1 requiring emergent surgical intervention. While CM-1 is generally a chronic and asymptomatic condition, our findings highlight that a subset of patients may experience life-threatening acute respiratory insufficiency, necessitating urgent decompressive surgery. The presence of syringomyelia appears to be a common feature in these cases, suggesting its potential role in the pathophysiology of acute deterioration. Our literature review further supports the idea that emergent surgical decompression remains the standard approach for managing acute CM-1 presentations, often leading to favorable outcomes, although long-term neurological impairments, such as persistent tetraparesis and ventilatory dependence, may persist in some patients. The identification of predictive risk factors for acute decompensation remains uncertain, emphasizing the need for further research to refine patient selection for early surgical intervention and optimize clinical management strategies. Given the rarity of acute CM presentations, continued case reporting and larger-scale studies are essential to improve our understanding of the condition's natural history and refine management approaches. Better recognition of high-risk patients could enable earlier interventions, potentially preventing catastrophic neurological and respiratory complications.
